# Efficacy and speed of kill of a topically applied formulation of dinotefuran-permethrin-pyriproxyfen against weekly tick infestations with *Rhipicephalus sanguineus* (*sensu lato*) on dogs

**DOI:** 10.1186/s13071-016-1561-y

**Published:** 2016-05-16

**Authors:** Jeffrey Blair, Josephus J. Fourie, Marie Varloud, Ivan G. Horak

**Affiliations:** Ceva Santé Animale, 10 Avenue de la Ballastière, 33500 Libourne, France; ClinVet International, P.O. Box 11186, Universitas, 9321 South Africa; Department of Veterinary Tropical Diseases, Faculty of Veterinary Science, University of Pretoria, Onderstepoort, 0110 South Africa

**Keywords:** Topical application, Acaricidal efficacy, Brown dog tick, Canine, Prevention

## Abstract

**Background:**

*Rhipicephalus sanguineus* (*sensu lato*) is a vector of canine babesiosis, anaplasmosis and ehrlichiosis. In order to reduce the chance of transmission of these diseases, an ectoparasiticide should rapidly repel or kill new infestations with this tick. The primary objective of the present study was to evaluate the treatment and preventive acaricidal efficacy of Vectra® 3D (54.45 mg/ml of dinotefuran, 396.88 mg/ml of permethrin and 4.84 mg/ml of pyriproxyfen) against *R. sanguineus* (*s.l*.) measured at 2, 8, and 48 h after treatment and weekly re-infestation.

**Methods:**

Twenty-four dogs were each infested with 50 adult *R. sanguineus* (*s.l*.) on Day -7 and allocated to three groups (*n* = 8) based on tick counts: an untreated control group (Group 1), and two groups (Groups 2 and 3) treated with Vectra®3D. The dogs in each group were infested with 50 ticks on Day -2. Vectra®3D was administered topically to the dogs on Day 0. Ticks were counted, *in situ* at 2 and 8 h after treatment on dogs in Groups 1 and 3. Group 3 was then withdrawn from the study and ticks were counted and removed from the dogs in Groups 1 and 2, 48 h after treatment. On Days 7, 14, 21, 28, 35 and 42, the dogs in Groups 1 and 2 were re-infested with 50 ticks, which were then counted *in situ* at 2 and 8 h, and counted and removed at 48 h after re-infestation.

**Results:**

Ticks from the initial infestation were visually unaffected by 2 and 8 h after treatment. However, by 2 h after weekly re-infestation the arithmetic mean (AM) efficacy of Vectra® 3D from Days 7 through 28 ranged from 61.1 to 78.8 %, falling to 60.1 and 47.4 % on Days 35 and 42 respectively. By 8 h after weekly re-infestation, the AM efficacy ranged from 89.1 to 97.4 % falling to 81.4 and 69.8 % on Days 35 and 42 respectively. The AM efficacy 48 h after treatment after the initial infestation was 22.9 % but after weekly re-infestation the efficacy at 48 h ranged from 89.1 to 100.0 %, falling to 86.0 and 81.1 % on Days 35 and 42 respectively.

**Conclusion:**

Vectra® 3D demonstrated significant efficacy against new infestations of adult *R. sanguineus* (*s.l*.) ticks within 2 h of infestation as compared to the untreated control group and achieved over 89.1 % efficacy within 8 h of infestation for up to 4 weeks after administration. These results indicate that Vectra® 3D has a rapid and significant efficacy against new infestations of adult *R. sanguineus* (*s.l*.) ticks and should therefore be considered as part of a strategy against important vector-borne diseases in dogs.

## Background

The topical formulation (Vectra® 3D, DPP) used in this study is a combination of 54.45 mg/ml of dinotefuran, 396.88 mg/ml of permethrin and 4.84 mg/ml of pyriproxyfen with a broad spectrum of activity against external parasites of dogs. Permethrin, the primary acaricidal component of this formulation, is a photostable synthetic pyrethroid with a relatively long residual activity that prevents the closure of the sodium channels, leaving the nerve cell membrane in a permanent state of depolarization [1]. It is this mode of action that results in the sudden “knock down” effect on pests and especially the “hot foot” reaction of ticks coming in contact with treated dogs. Moreover, permethrin is also an arthropod repellent [2]. Dinotefuran is a fast-acting insecticide furanicotinyl belonging to the most recent generation of neonicotinoid [3] and pyriproxyfen is an insect growth regulator that targets and disrupts the reproductive and endocrine systems of insects [4].

*Rhipicephalus sanguineus* (*sensu lato*) is a three-host tick species, and with few exceptions its larvae, nymphs and adults feed almost exclusively on domestic dogs [5–8]. It is the most widespread tick in the world [6]. Female ticks may deposit eggs under a dog’s bedding or in nearby sheltered spots, or they may crawl up surrounding structures and lay eggs in cracks and crevices in these structures, which may also be used by the larvae and nymphs [6, 9]. Dogs that are caged, chained or kennelled may become particularly heavily infested [8, 10] and all stages of development can simultaneously be present on the same dog [10, 11].

Ticks are vectors of many bacterial and protozoal diseases in dogs. *R. sanguineus* (*s.l*.) has been confirmed or implicated as the vector of the bacterial agents *Ehrlichia canis, Anaplasma platys, Rickettsia rickettsii,* and *Rickettsia conorii* and the protozoal organisms *Babesia vogeli* and *Hepatozoon canis* [12]*.* The two most important diseases in dogs caused by organisms transmitted by *R. sanguineus* (*s.l*.) are canine monocytic ehrlichiosis caused by *E. canis* and canine babesiosis caused by *B. vogeli* [7]. Although data about the minimal time required for transmission of these pathogens are scarce, it is accepted that the time required to transmit these two diseases is very different. Transmission of protozoan parasites like *Babesia* protozoa generally requires at least 24 to 48 h after tick attachment, in order for their sporoblasts to mature into sporozoites in the salivary glands of the tick [13, 14]. In contrast, bacterial pathogens such as *E. canis* are transmitted by *R. sanguineus* (*s.l*.) much more quickly - within a few hours after attachment [15]. Consequently, in order to significantly reduce the risk of tick-borne pathogens a product must demonstrate a rapid onset of acaricidal and/or repellent activity, preferably within a few hours.

The primary objective of the present study was to evaluate the curative and preventive acaricidal efficacy of a DPP combination against *R. sanguineus* (*s.l*.) measured at 2, 8, and 48 h after treatment and after weekly re-infestation.

## Methods

The study was a parallel group, blinded, randomized, single centre, controlled efficacy study. The study was conducted by an independent contract laboratory facility in South Africa in accordance with the International Cooperation on Harmonisation of Technical Requirements for Registration of Veterinary Medicinal Products (VICH) guideline 9 entitled ‘Good Clinical Practice’. All procedures were in compliance with South African Animal Welfare Act Regulations ‘The care and use of animals for scientific purposes’ and the protocol was approved by the local animal ethics committee.

The 24 dogs enrolled in the investigation were mongrels of both sexes, older than six months, and weighed between 10.4 and 22.8 kg. All dogs were dewormed prior to the start of the study and were acclimatized to the kennel environment for seven days before treatment. The animals were housed individually for the duration of the study in an indoor/outdoor run that conformed to accepted animal welfare guidelines, and no physical contact between dogs was possible. They were fed once a day according to the food manufacturer’s recommendations, and water was available *ad libitum*.

The study design is summarized in Table 1. A laboratory-bred strain (U.S. origin) of *R. sanguineus* (*s.l*.) was used throughout the investigation. Ticks used for all infestations were unfed, at least one week old and had a balanced sex ratio (50 % female: 50 % male). Seven days before treatment all the dogs were infested with 50 adult *R. sanguineus*. Forty-eight hours after infestation the ticks were counted and removed and the dogs were ranked within sex in descending order of individual pre-treatment tick counts and subsequently blocked into eight blocks of three animals each. From each block, dogs were randomly allocated to three groups of eight and the groups were coded to blind the investigators performing the post-treatment assessments. All dogs were infested on Day -2 and dogs in Groups 2 and 3 were treated on Day 0 while dogs in Group 1 served as untreated controls. *In situ* counts were performed on dogs in Groups 1 and 3 on the day of treatment and thereafter the dogs in Group 3 were withdrawn from the study. The dogs in Group 3 were included in the study because of the possibility of unintentional manual removal of the DPP formulation while the product was still drying during the *in situ* tick counts performed at 2 h after administration. Dogs in Groups 1 and 2 continued in the study with infestations performed at weekly intervals from Day 7 through 42. Ticks on the dogs in Groups 1 and 2 were counted *in situ* at 2 h and 8 h after each weekly re-infestation and counted and removed 48 h after treatment on Day 0 and after each weekly re-infestation from Day 7 to Day 42 (Table 1). Ticks that were removed 48 h after treatment or re-infestation were categorized according to their attachment, engorgement and viability status at the time of removal according to the parameters listed in Table 2 [16].

**Table 1:** Design of a study to determine the efficacy and speed of kill of DPP against adult *R. sanguineus* s.l

Day	Procedure
-14 to -7	Acclimatization to kennel environment
-7	Infestation of all dogs with 50 adult ticks
-5	Tick counts and allocation of dogs to three groups of eight
-2	Infestation of dogs with 50 adult ticks
0	Topical application of DPP to dogs in Groups 2 and 3; *in situ* tick counts on dogs in Groups 1 and 3 at 2 and 8 h after treatment
1	Removal of ticks from dogs in Group 3 and withdrawal of this group of dogs from the study
2	Tick counts and tick removal on dogs in Groups 1 and 2
7, 14, 21, 28, 35, 42	Infestation of dogs in Groups 1 and 2 with 50 adult ticks; *in situ* tick counts on dogs in Groups 1 and 2 at 2 and 8 h after infestation
9, 16, 23, 30, 37, 44	Tick counts and tick removal on dogs in Groups 1 and 2, 48 h after infestation

**Table 2:** Status of adult *R. sanguineus* (*s.l*.) removed from dogs 48 h after treatment with DPP on Day 0 and after weekly re-infestation from Day 7 to Day 42

Category	Condition^a^	Attachment status
1	Live	Unattached
2	Live	Attached, unengorged^b^
3	Live	Attached, engorged^c^
4	Dead	Unattached
5	Dead	Attached, unengorged
6	Dead	Attached, engorged

^a^Live/Dead status determined by observation for movement after stimulus with a probe or gentle CO_2_ exposure

^b^Unengorged = No filling of the alloscutum

^c^Engorged = obvious or conspicuous filling of the alloscutum

Treatment was administered by parting the hair and applying the appropriate volume (3.6 ml) of DPP directly onto the skin in a continuous line from the base of the tail along the middle of the back to between the shoulder blades, according to the label instructions. The time at which treatment was administered to each animal and the time at which it was infested with ticks were recorded. This was done to ensure that *in situ* counting of ticks 2 h (± 5 min) or 8 h (± 30 min) after treatment or re-infestation, and counting and removal of ticks 48 h (± 2 h) after treatment or re-infestation were accomplished as close as possible to the specified target times. During *in situ* counts, ticks were found by direct observation following parting of the hair and by palpation. During removal counts the same procedure was followed but ticks were removed upon counting and the dogs were also combed to ensure that all ticks had been counted and removed.

The primary assessment criteria was the number of ticks counted on the control and the treated groups of dogs on the various assessment times and days, with efficacy calculations based on geometric (GM) and arithmetic (AM) means. Geometric means were calculated using the tick count data + 1, and 1 was subsequently subtracted from the result to obtain a meaningful mean value for each group. Efficacy of the DPP formulation against adult *R. sanguineus* (*s.l*.) at 2, 8 and 48 h after treatment or infestation was calculated as follows:

$$ \mathrm{Efficacy}\ \left(\%\right) = 100 \times \left({\mathrm{M}}_{\mathrm{c}}\hbox{--}\ {\mathrm{M}}_{\mathrm{t}}\right)\ /\ {\mathrm{M}}_{\mathrm{c}} $$

where:

- M_c_ = Mean number of live ticks (categories 1, 2, 3 and 6) on dogs in the untreated control group (Group 1) at a specific time point.
- M_t_ = Mean number of live ticks (categories 1, 2, 3 and 6) on dogs in the treated groups (Groups 2 and 3) at a specific time point.

Comparisons of tick counts between groups were conducted using a one-way ANOVA with an administration effect (*P* < 0.05). In addition, the groups were compared by a non-parametric analysis using the Mann-Whitney test on untransformed tick counts. Ticks in category 6 were included in the theoretical calculation because, if found, these ticks would have succeeded in engorging before they were killed. In this study, however, there were no ticks classified as category 6.

## Results

**Fig. 1:**
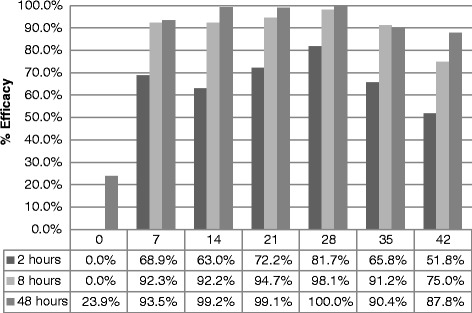
Summary of calculated geometric mean efficacy of DPP against adult *R. sanguineus* (*s.l.*)

The GM number of ticks on the eight dogs in the untreated control group varied between 24.0 and 31.5 in the counts conducted 48 h after treatment or weekly re-infestation, demonstrating that an adequate level of infestation was achieved in the control group. The efficacy of a single topical application of DPP against adult *R. sanguineus* (*s.l*.) 2, 8 and 48 h after treatment or weekly re-infestation is summarized in Fig. 1. Efficacy against a well established infestation was not demonstrated at 2, 8 and 48 h after treatment in this study. However, after weekly re-infestation on Days 7, 14, 21, 28, 35 and 42, the efficacy calculated with GM for the 2 h counts was 68.9, 63.0, 72.2, 81.7, 65.8 and 51.8 %, respectively (Table 3). By 8 h after infestation efficacy had increased to 92.3, 92.2, 94.7, 98.1, 91.2 and 75.0 % on Days 7, 14, 21, 28, 35 and 42, respectively (Table 4). At 48 h after infestation on Days 7, 14, 21, 28, 35 and 42 the calculated efficacies were 93.5, 99.2, 99.1, 100.0, 90.4 and 87.8 %, respectively (Table 5). There was a significant difference in all counts conducted after Day 7 between the control and the treated groups (*P* < 0.005).

**Table 3:** Mean tick counts and percent efficacy of DPP at 2 hours after treatment or infestation against adult *R. sanguineus* (*s.l*.)

Day	0^c^	7	14	21	28	35	42
AM^a^ tick counts control	28.1	39.1	38.1	37.6	38.3	41.0	38.8
AM tick counts treated	34.3	14.1	14.6	14.6	8.1	16.4	20.4
AM % efficacy	0	63.9^d^	61.6^d^	61.1^d^	78.8^d^	60.1^d^	47.4^d^
Test *F*_(1,14)_	–	72.65	94.65	36.77	151.08	51.15	26.28
*P*-value	–	< 0.0001	< 0.0001	< 0.0001	< 0.0001	< 0.0001	0.0002
GM^b^ tick counts control	27.7	38.8	37.8	37.5	37.9	40.8	38.4
GM tick counts treated	33.9	12.1	14.0	10.4	7.0	14.0	18.5
GM % efficacy	0	68.9^d^	63.0^d^	72.2^d^	81.7^d^	65.8^d^	51.8^d^
Test *F*_(1,14)_	–	21.05	63.40	11.78	53.82	19.99	15.05
*P*-value	–	0.0004	< 0.0001	0.004	< 0.0001	0.0005	0.0017

^a^ *AM* arithmetic mean

^b^ *GM* geometric mean

^c^Counts obtained from Group 3 dogs

^d^Significant difference between treated and control group (*P* <0.05) based on ANOVA

**Table 4:** Mean tick counts and percent efficacy of DPP at 8 hours after treatment or infestation against adult *R. sanguineus* (*s.l*.)

Day	0^c^	7	14	21	28	35	42
AM^a^ tick counts control	25.0	34.3	27.6	35.3	33.9	29.5	37.6
AM tick counts treated	29.1	3.8	2.9	2.9	0.9	5.5	11.4
AM % efficacy	0.0	89.1^d^	89.6^d^	91.8^d^	97.4^d^	81.4^d^	69.8^d^
Test *F*_(1,14)_	–	173.07	189.78	443.41	302.28	41.35	73.68
*P*-value	–	< 0.0001	< 0.0001	< 0.0001	< 0.0001	< 0.0001	< 0.0001
GM^b^ tick counts control	24.7	33.9	27.3	35.1	33.5	28.7	37.3
GM tick counts treated	28.5	2.6	2.1	1.8	0.6	2.5	9.3
GM % efficacy	0.0	92.3^d^	92.2^d^	94.7^d^	98.1^d^	91.2^d^	75.0^d^
Test *F*_(1,14)_	–	60.12	73.41	69.47	219.96	22.84	27.16
*P*-value	–	< 0.0001	< 0.0001	< 0.0001	< 0.0001	0.0003	0.0001

^a^ *AM* arithmetic mean

^b^ *GM* geometric mean

^c^Counts obtained from Group 3 dogs

^d^Significant difference between treated and control group (*p* <0.05) based on ANOVA

**Table 5:** Mean tick counts and percent efficacy of DPP at 48 hours after treatment or infestation against adult *R. sanguineus* (*s.l.*)

Day	2	9	16	23	30	37	44
AM^a^ tick counts control	27.9	26.4	30.8	31.9	30.8	31.3	24.5
AM tick counts treated	21.5	2.9	0.4	0.5	0.0	4.4	4.6
AM % efficacy	22.9	89.1^c^	98.8^c^	98.4^c^	100.0^c^	86.0^c^	81.1^c^
Test *F*_(1,14)_	–	39.81	83.96	271.72	159.73	55.15	60.79
*P*-value	–	< 0.0001	< 0.0001	< 0.0001	< 0.0001	< 0.0001	< 0.0001
GM^b^ tick counts control	27.0	24.9	29.7	31.5	30.1	30.0	24.0
GM tick counts treated	20.5	1.6	0.3	0.3	0.0	2.9	2.9
GM % efficacy	23.9	93.5^c^	99.2^c^	99.1^c^	100.0^c^	90.4^c^	87.8^c^
Test *F*_(1,14)_	–	40.64	321.09	286.29	1, 964.66	34.60	26.80
*P*-value	–	< 0.0001	< 0.0001	< 0.0001	< 0.0001	< 0.0001	0.0001

^a^ *AM* arithmetic mean

^b^ *GM* geometric mean

^c^Significant difference between treated and control group (*P* <0.05) based on ANOVA

## Discussion

One of the most important aspects of a rapid speed of kill for an acaricide is the prevention of tick-transmitted diseases. In order to achieve this goal an acaricide should prevent the attachment of ticks or rapidly kill them as soon as they access the dog. Compliantly with previous experiment [9], therapeutic efficacy against an existing infestation was not demonstrated in this study. However, high levels of preventive efficacy were quickly achieved (Fig. 1; Table 3). By 2 h after infestation there were significantly fewer ticks on treated dogs than on the controls. The discrepancy between the therapeutic and preventive efficacy can be explained by the time required for the formulation to spread over the body of the dog and also by the fact that permethrin has both a direct killing effect and an important repellent activity that prevents ticks attaching to the dog and start feeding [2, 9].

By Day 7 after treatment the active ingredients had spread throughout the hair-coat and on the skin and between 63 and 81.7 % of ticks were killed within 2 h after being released onto dogs from Day 7 through Day 35, dropping to 51.8 % on Day 42 (it should be noted that the DPP formulation is labelled for monthly re-application). The rapid acaricidal efficacy observed at 2 h increased to > 90 % by 8 h after each infestation from Day 7 through Day 35. The acaricidal efficacy recorded 48 h after infestation was in concordance with previous measurements performed against *R. sanguineus* (*s.l*.) adult ticks with the same product for 1 month after treatment [9]. In the present experiment, residual efficacy was assessed for 6 weeks and was above 90 % (GM) for 5 weeks.

The earliest speed of kill data against *R. sanguineus* (*s.l*.) reported after administration of a permethrin-based combination product on dogs were recorded 3 h after weekly infestation and efficacy varied from 69.9 to 88.1 % between days 7 and 28 after treatment [17]. The acaricidal efficacy at 2 h and strong level of prevention of re-infestation by 8 h is an important finding in that it has been demonstrated that *E. canis* can be transmitted as early as 3 h after exposure to infected *R. sanguineus* ticks [15]. Afoxolaner, a systemically active isoxazoline recommended for monthly oral treatment, has been shown to have therapeutic acaricidal efficacy of 93 % by 12 h after treatment. However, preventive efficacies were below 77 % at 12 h against weekly infestations with *I. ricinus* from Day 7 through Day 28 [18] and between 0 and 14 % at 8 h against weekly infestations with *R. sanguineus* from Day 7 through Day 35 after treatment [19]. Fluralaner, also a systemically active isoxazoline recommended for oral administration once every 3 months, demonstrated a therapeutic efficacy of 97.9 % against infestations with *I. ricinus* by 8 h after treatment, and had preventive efficacy of 96.8 % by 8 h over the first 4 weeks after treatment. However, efficacy declined to 83.5 and 45.8 % at weeks 8 and 12, respectively [20]. In a comparative efficacy study of topical DPP, oral fluralaner and oral afoxolaner that measured their preventive acaricidal efficacy against *R. sanguineus* at 12 h, the topical formulation demonstrated 77–98 % efficacy compared to 21–49 % for afoxolaner and 58–89 % for fluralaner over a period of one month [21]. In another study, in which acaricidal efficacy was measured at 3 h after infestation with *R. sanguineus*, the efficacy of afoxolaner varied between zero and 26 % and of fluralaner between 13 and 53 %, and the tick counts of neither group were significantly different from those of the negative control group at this time interval [22].

The rapid preventive acaricidal efficacy of the topical formulation of DPP against new infestations of *R. sanguineus*, could potentially reduce the likelihood of the transmission of *E. canis* to dogs by infected ticks. Moreover, the sustained rapid preventive acaricidal efficacy would also reduce the risk of dogs becoming infected with other tick-borne diseases such as babesiosis associated with *R. sanguineus*. Regular monthly administration of DPP would also prevent re-infestation by ticks and prevent the development of local foci of tremendous numbers of ticks so often associated with *R. sanguineus* (*s.l*.). At localities where levels of infestation are particularly severe, in addition to treating the dog with DPP, the environment should be thoroughly searched for ticks which can then be eradicated by the application of a suitable acaricide formulated for this purpose. It is wise to remember that there are always many more free-living ticks within the dog’s environment than on the dog itself [6, 7].

## Conclusion

This study demonstrated the high level of speed of kill of DPP against new infestations of *R. sanguineus* (*s.l*.) ticks on dogs. Topical treatment with the formulation reached or exceeded 90 % efficacy within 8 h of infestation and a significant number of newly acquired ticks were killed within 2 h of infestation for up to 5 weeks after administration. Monthly administration of this formulation can be considered as a reliable tool for protection against ticks and also likely for diseases they can transmit to dogs.
